# Xanthogranuloma in the heavily irradiated low neck in a patient with head and neck cancer

**DOI:** 10.1186/s40463-016-0134-6

**Published:** 2016-03-24

**Authors:** Lisa Singer, Sarah M. Calkins, Andrew E. Horvai, William R. Ryan, Sue S. Yom

**Affiliations:** Department of Radiation Oncology, University of California, San Francisco, 1600 Divisadero St, Suite H-1031, San Francisco, CA 94143 - 1708 USA; Department of Pathology, University of California, San Francisco, San Francisco, CA USA; Department of Otolaryngology, University of California, San Francisco, 1600 Divisadero St, Suite H-1031, San Francisco, CA 94143 - 1708 USA

**Keywords:** Xanthogranuloma, Radiation therapy, Head and neck cancer, Skin, Toxicity

## Abstract

**Background:**

Head and neck cancer is often managed with a combination of surgery, radiation therapy, and chemotherapy, and skin toxicity is not uncommon. Xanthogranuloma is a pathological finding resulting from an inflammatory reaction that has not been previously reported following head and neck radiation therapy.

**Case presentation:**

A patient with squamous cell carcinoma of the oropharynx, treated with definitive chemoradiation and hyperthermia, presented at eight-month follow-up with an in-field cutaneous lesion in the low neck, initially concerning for recurrent tumor.

Biopsy showed xanthogranuloma and the patient underwent complete resection with congruent surgical pathology. The patient remained free of malignancy but continued to experience wound healing difficulties at the resection site which resolved with specialized wound care and hyperbaric oxygen.

**Conclusions:**

Skin toxicity is not uncommon in patients with head and neck cancer treated with radiation therapy. Awareness of unusual pathologic sequelae, such as xanthogranuloma, is needed to provide patient counseling while continuing appropriate surveillance for recurrent malignancy.

## Background

For patients with locally advanced head and neck cancer, the use of radiation therapy with concurrent chemotherapy is supported by multiple randomized trials [1]. This treatment can produce both acute and late toxicity to normal tissues [2]. Known late toxicities to the skin include permanent skin tanning, telangiectasias, necrosis, and fibrosis.

We report a case of a patient with head and neck cancer treated with radiation therapy who later presented with xanthogranuloma arising within the radiation field. Xanthogranuloma is a histopathological diagnosis, referring to a lesion comprised of abundant histiocytes, often displaying foamy cytoplasm and associated foreign body giant cells [3]. Juvenile xanthogranulomatosis (JXG) is a clinical diagnosis first described in the 1950s, referring to children with one or more of these lesions [4]. JXG is thought to be a benign disorder, although ocular involvement may impact 0.5 % of patients with skin involvement [4]. Xanthogranulomas have been described in adults in association with multiple possible risk factors, including trauma, infection, and malignancy. Xanthogranuloma has been reported in a patient treated with radiation therapy for breast cancer, but it has not been previously reported in the English literature following head and neck irradiation [5]. To increase awareness of this potential late effect of chemoradiation, we present the case and review relevant related literature.

## Case presentation

At the age of 45, a patient with a 30-pack year history of tobacco use and no significant past medical history presented to our institution with pathology-proven AJCC Stage IVA, T3 (lingual epiglottis extension), N2c (bilateral lymph node involvement), M0 squamous cell carcinoma of the left base of tongue. Notably, he had history of a palpable left upper neck mass three years prior, but fine needle aspiration (FNA) at that time was non-diagnostic. The neck mass recurred and he initially pursued treatment with homeopathic therapy. The mass increased in size, leading him to seek further medical attention and ultrasound showed a very large left supraclavicular mass and a right submandibular mass. FNA of the left neck mass showed p16-overexpressing squamous cell carcinoma. Positron emission tomography with computed tomography (PET/CT) showed a mass involving the left tonsillar pillar and extending to the piriform sinus, with increased avidity on FDG-PET and measuring over 3 cm in longest diameter. He was also noted to have FDG-avid suspicious lymph nodes involving the right level IIA, left level IIA and left level IV. A left parapharyngeal lymph node was also concerning. At the time of the initial radiation oncology consult he endorsed weight loss, left neck pain, dysgeusia, decreased appetite, and tracheal pressure without shortness of breath. He had received no treatment for the left oropharyngeal cancer except for treatment with hyperthermia in ten fractions at an outside facility.

On exam, he was found to have Karnofsky Performance Status of 80, vital signs within normal limits, and a greater than 6 cm left-sided lymph node conglomerate and palpable left level II lymph node and right level II/III lymph nodes, with slight tracheal deviation to right. Concurrent cisplatin-based chemoradiation was advised and the patient consented to treatment.

### Treatment

The patient was treated at our institution with intensity-modulated radiation therapy, 70.88 Gy in 34 fractions, prescribed to the 88 % isodose line, targeting the left oropharynx and bilateral neck with 6 MV photons, with concurrent hyperthermia given twice a week to the massive nodal mass as well as cisplatin.

His treatment course was complicated by intractable nausea and vomiting following the first cycle of cisplatin. Due to these symptoms, as well as purulent drainage from the left neck mass following radiation, he was hospitalized. There was no evidence of infection identified during hospitalization but there was a golf-ball-sized area of skin rupture in the left low neck emitting pus and drainage that was attributed to tumor necrosis. His chemotherapy was switched to weekly administration. Over the course of treatment, he also developed grade 2 oral mucositis and dry desquamation of other parts of the skin. Due to a clinically evident decrease in tumor size, a repeat radiation planning CT was obtained and a new radiation plan was generated for use during the last ten fractions. The patient did not require a feeding tube during treatment, although for the month following completion of radiation therapy, he elected to receive frequent intravenous hydration.

### Post-treatment surveillance

At the six month follow-up appointment, magnetic resonance imaging (MRI) of the head and neck showed treatment response of the primary tumor and bilateral neck. On interval physical exam, he had residual right level II lymphadenopathy and stiffness in the left neck with erythematous and hypo-pigmented areas. There were no palpable base of tongue lesions. At 3 month follow-up, PET/CT showed that the right level II lymph node was not FDG-avid. At repeat MRI two months later, the right level II lymph node was stable and the patient appeared to have no evidence of disease although on exam, telangiectasias were noted in the skin at the left supraclavicular region.

**Fig. 1:**
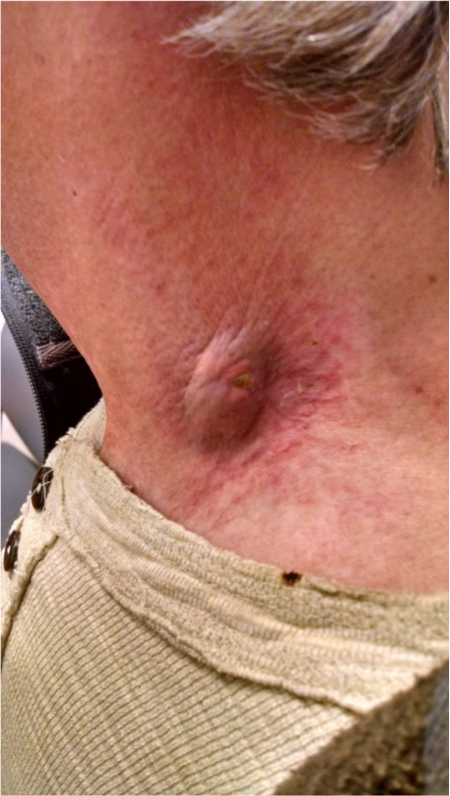
Left low neck lesion in patient treated with chemoradiation for head and neck cancer

At follow-up eight months after completion of chemoradiation, he was found to have a 1.5x1.5 cm irregularly shaped, reddish-purplish-colored lesion extruding out from the skin in level IV of the left neck, with surrounding deep red ecchymosis. This new mass was located in the area of the rupture through skin. MRI at that time showed small left level 3, 5a, and 5b lymphadenopathy underlying the area. The cutaneous left neck lesion was concerning for cancer recurrence and he was referred for biopsy. The lesion is shown in Fig. 1.

### Cytopathology

FNA with three passes was performed. Aspirate material was abundantly cellular and composed of numerous foamy histiocytes and a sparse inflammatory infiltrate within a background of lipid debris. No epithelial cells were noted on aspirate smears or cell block material. Additionally, a pankeratin immunohistochemical stain was performed and was negative, further arguing against an epithelial component.

CT of the chest was also obtained, which revealed no metastases. The patient was referred to otolaryngology-head and neck surgery for management of the left neck lympadenopathy. Flexible fiberoptic nasopharyngolaryngoscopy was normal. A left level IV lesion was again visible and palpable on exam, with surrounding skin intact but with associated erythema. Punch biopsy of the cutaneous lesion was performed showing dystrophic xanthomatosis and resection was advised. MRI was re-reviewed and it was determined that there was no clear sign of cancer persistence or recurrence within the deep neck.

### Resection of xanthogranuloma

At nine months following completion of chemoradiation, the patient elected for gross resection of the left neck mass. The mass was over 4 cm in size, and transfer of adjacent skin that had not been radiated was required to close the defect.

### Surgical pathology

**Fig. 2:**
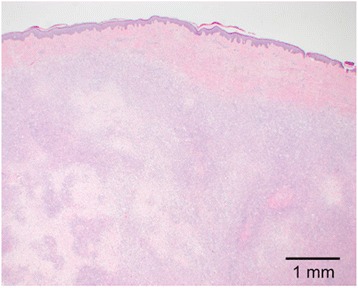
Histologic section showing dense population of foamy histiocytes (xanthoma cells), occasional multinucleate giant cells, and scattered interspersed lymphocytes involving the deep dermis and subcutis

**Fig. 3:**
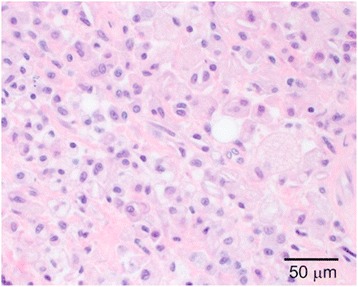
Histologic section showing foamy histiocytes, multinucleate giant cells, and interspersed lymphocytes

**Fig. 4:**
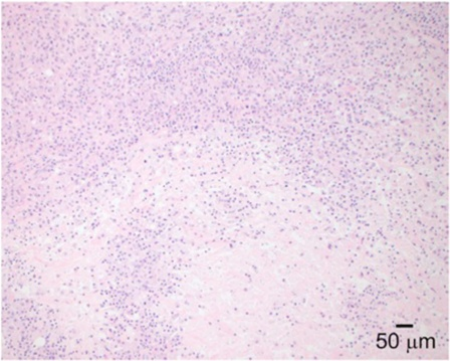
Histologic section showing degenerating collagen and nuclear debris

**Fig. 5:**
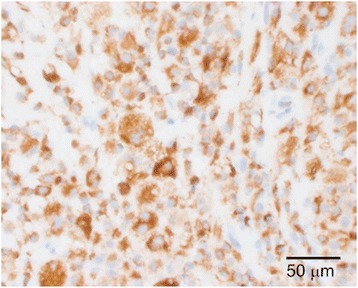
Histochemical staining of CD68 in foamy cells, supporting a histiocyte lineage.

Gross examination revealed a yellow, firm, oval subcutaneous mass measuring 2.1 x 1.6 x 1.0 cm. Histologic sections revealed a dense population of foamy histiocytes (xanthoma cells), occasional multinucleate giant cells and scattered interspersed lymphocytes (Figs. 2 and 3) involving the deep dermis and superficial subcutis. In some areas, these cells were found to surround degenerating collagen and nuclear debris (Fig. 4). No obvious epithelial cells or carcinoma were identified on H&E sections and a keratin immunostain was negative, further arguing against a component of metastatic carcinoma. The foamy cells were positive for CD68, supporting a histiocyte lineage (Fig. 5). Histochemical stains were negative for acid-fast and fungal microorganisms, arguing against an infectious component. Lack of immunostaining for S100-protein and SOX10 argued against the possibility of melanoma. Overall, the findings were consistent with dystrophic xanthomatosis (also known as dystrophic xanthogranulomatosis).

### Surveillance

**Fig. 6:**
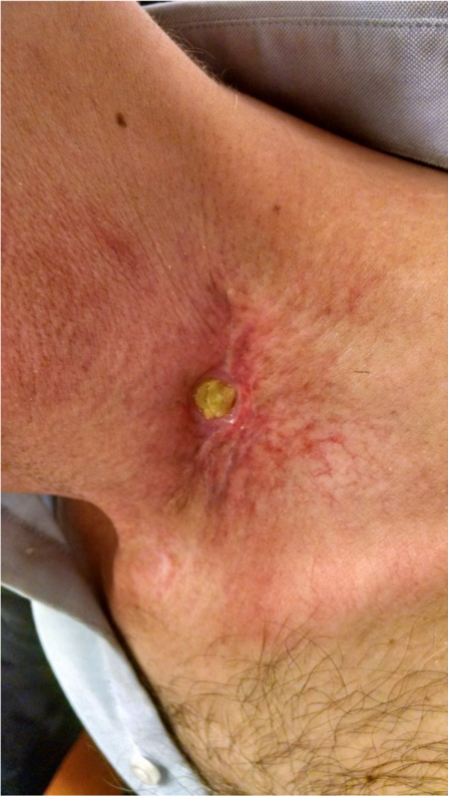
Left low neck with nonhealing wound after resection of xanthogranuloma

**Fig. 7:**
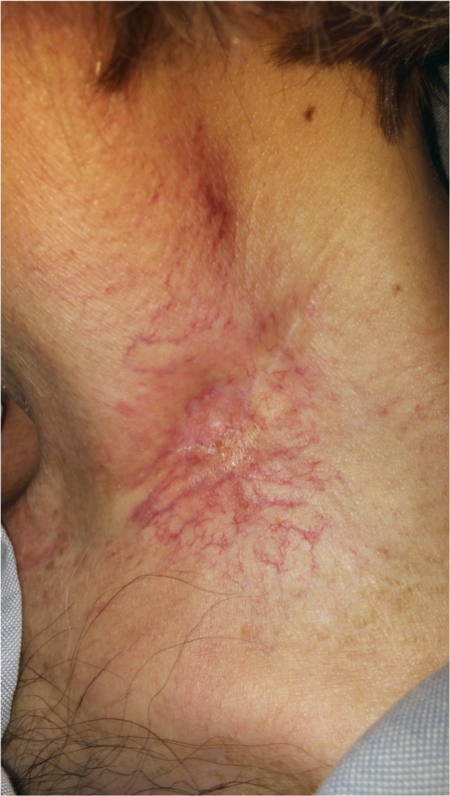
Resolution of left low neck wound after xanthogranuloma resection

MRI seven weeks after resection showed no evidence of disease. Due to the patient’s history of lymphocytopenia pre-dating initiation of chemotherapy and possible association between the development of xanthogranulomas and hematological malignancy, the patient was re-evaluated by the hematology service [6]. The prior low lymphocyte count was thought to be spurious and no evidence for a lymphoproliferative disorder was identified. At three months following the resection, the patient was found to have mild erythema of the left neck with an open lesion at the post-operative site (Fig. 6). Medical grade honey and foam dressing were prescribed, with continued follow-up to monitor wound healing. At 14 months from chemoradiotherapy completion and 6 months from lesion excision, the patient had no sign of cancer recurrence but continued to experience wound healing difficulties. It was decided to administer a month of hyperbaric oxygen treatments and collagenase dressings. By the next followup 3 months later, the skin had finally closed, leaving residual fibrosis and telangiectasia (Fig. 7).

## Conclusions

At eight months following completion of chemoradiation for a locoregionally advanced head and neck cancer, a patient was to found to develop a xanthogranuloma within the radiation field. The xanthogranuloma arose in a location where the skin had ruptured over a large nodal mass during the course of treatment. This represents a late radiation toxicity that has not yet been reported in the English literature in a patient with head and neck cancer. Assessment of patient and treatment-related factors may improve understanding of this toxicity.

A recent consensus report discussed factors contributing to skin toxicity in patients with head and neck cancer treated with chemoradiation [7]. Host factors contributing to toxicity include nutritional status, sun exposure, and smoking, as well as comorbidities such as lupus, diabetes, or hypertension. While this patient did not have known comorbidities, he did have a history of smoking. His nutritional status was reasonably maintained during treatment. Treatment-related factors cited in the consensus report included skin dose and fraction size >2 Gy per fraction, as well as the use of bolus and radio-sensitizing therapies. Due to the extensive nodal disease in the low left neck, the skin dose was high and this increased the risk for toxicity. This patient was also treated with two radio-sensitizing therapies in the area: cisplatin and hyperthermia.

Prior studies on the use of radiation, chemotherapy, and hyperthermia in patients with head and neck cancer have not identified the skin toxicity that we describe in this case report. In a study of 40 patients followed for a median of 9 months, two Grade 1 burns were identified [8]. In a retrospective study of radiation with or without hyperthermia, acute toxicity was similar and late toxicity was higher in the combined treatment group, with two patients diagnosed with bone necrosis [9]. A Phase I-II study on the use of concurrent cisplatin, hyperthermia and radiation [10] showed that acute skin toxicity was slightly higher with the use of hyperthermia than without (dry desquamation in >50 % of skin surface area vs <50 % in the areas not treated with hyperthermia). Late effects were similar with and without hyperthermia, although one case of RTOG/EORTC grade 4 subcutaneous necrosis was identified within the hyperthermia field. In a single-institution study randomizing patients with superficial tumors to radiation with or without hyperthermia, the incidence of thermal burns was increased with hyperthermia [11]. In summary, this information suggests that the radio-sensitizing effects of hyperthermia may put patients at higher risk for toxicity and should be monitored carefully.

Xanthogranuloma has been reported in a patient receiving breast radiation [5]. In this case report, the lesion was identified 12 months following completion of breast radiation therapy. The authors speculated that due to the time course, radiation likely played a role. Unlike the xanthogranuloma in our patient, this lesion was 4 mm in maximum diameter and was able to be excised with punch biopsy. Both the breast cancer patient and our patient received radiation therapy and chemotherapy, although, as per standard of care, the treatments were not concurrent in the breast cancer patient. Our patient, like the breast cancer patient, presented with xanthogranuloma as a delayed late effect. However, in our patient, the tumor rupture through the skin during treatment likely contributed to chronically impaired integrity of the tissues in that region with a much more exuberant presentation of xanthogranuloma. We hypothesize that the necrosis and pus ignited a chronic inflammatory response arising preferentially in that location, releasing a macrophage flood that enabled development of xanthogranuloma.

While patients in the literature have been treated with chemoradiation and hyperthermia for head and neck cancer and have not developed xanthogranuloma, our patient is unique in that he received neoadjuvant hyperthermia at an outside facility prior to initiating definitive treatment at our institution. It is possible that the xanthogranuloma development was encouraged in part due to an interaction between this patient’s immune response and the intensive radio-sensitization resulting from concurrent cisplatin and localized hyperthemia. In any case, awareness of acute and late skin toxicities, including xanthogranuloma, can help direct the workup, which can be confusing given the overriding concern for malignant recurrence.

### Consent

Written informed consent was obtained from the patient for publication of this report and any accompanying images. The patient was given access to this report.
